# Effect of a Type II Collagen Fragment on the Expression of Genes of the Extracellular Matrix in Cells of the Intervertebral Disc

**DOI:** 10.2174/1874325000802010001

**Published:** 2008-01-23

**Authors:** F Mwale, H.T Wang, D.J Zukor, O.L Huk, A Petit, J Antoniou

**Affiliations:** Division of Orthopaedic Surgery, McGill University, Lady Davis Institute for Medical Research, SMBD-Jewish General Hospital, 3755, Chemin de la Cote Ste-Catherine, Montreal, QC H3T 1E2, Canada

## Abstract

Knowledge of factors regulating the turnover, repair, and degeneration of the intervertebral disc (IVD) is lacking. Although type II collagen (CII) fragments accumulate in the degenerative IVD, little is known of how they affect the degenerative process. A better understanding of the cellular interactions with fragments of matrix molecules are a key factor in promoting therapies for degenerative disc diseases. In the present study, we have investigated the effect of the CII (245-270) peptide on the expression of matrix molecules, proteinases, and interleukin genes in cells of the IVD. Cells isolated from the nucleus pulposus (NP) and annulus fibrosus (AF) of adult bovine tails were cultured up to 8 days in the absence (control) or presence of the CII (245-270) peptide. RT-PCR was used to analyze the expression of the different genes. Exposure of these cells to the CII (245-270) peptide led to a transient up-regulation of the aggrecan gene in AF cells while this up-regulation was maintained for a longer time in NP cells. The fragment also enhanced a transient up-regulation of the type II collagen gene in AF cells but had no effect in NP cells. The peptide enhanced transiently the expression of matrix metalloproteinase (MMP)-1 and cathepsin K genes in both AF and NP cells whereas it increased MMP-13 expression only in NP cells. The peptide up-regulated tissue inhibitor of metalloproteinase (TIMP)-1, TIMP-2, and TIMP-3 gene expression on day 1 in AF cells but had very little effect on their expression in NP cells. Finally, the CII (245-270) peptide had no effect on IL-6 expression while IL-1α was not expressed in these cells. In conclusion, our results showed that the CII (245-270) peptide differentially alter the expression of genes in bovine AF and NP cells and suggest that degradation products of collagen may be involved in the regulation of IVD homeostasis.

## INTRODUCTION

Low back pain is the most prevalent of musculoskeletal clinical conditions. It affects nearly everyone at some point in time and about 4-33% of the population at any given point [1]. Although the etiology of low back pain is often unclear, it is believed that intervertebral disc (IVD) degeneration plays a major role as it increases steeply with age, so that 60% of 70-year old discs are severely degenerated [2]. Though the majority of people affected will not require prolonged medical care or absence from work, about one third will require extensive care involving hospitalization [1]. Disorders of the lumbar spine that require surgical intervention include herniated IVDs, spinal stenosis, degenerative spondylolisthesis, degenerative scoliosis, and degenerative disc diseases [3, 4]. While present management of IVD pathology has been focused on symptoms associated with degeneration, much less study has been devoted to the mechanism of disc degeneration.

IVDs allow bending and twisting of the spine whilst resisting compression from gravity and muscle action [5, 6]. They are composite structures of the peripheral annulus fibrosus (AF) enclosing the central nucleus pulposus (NP). Their development is complex, involving several different connective tissue types [6]. The ability of IVD tissue to resist compressive forces is largely due to their high content of the proteoglycan aggrecan. However, IVD proteoglycans undergo changes in their metabolism and composition with ageing [7]. Thus, the reduction of aggrecan content observed with age within the disc may be the result of a decreased synthetic activity or an increased degradative capacity of the IVD cells.

Degeneration of the IVD results from excessive activity of tissue proteinases which degrade collagens of the extracellular matrix (ECM) [8-14]. Matrix metallo-proteinases (MMPs) are a family of nine or more highly homologous Zn(++)-endopeptidases that collectively cleave most, if not all, of the constituents of the ECM [8-10]. They have been implicated in the excessive breakdown of ECM components during IVD degeneration [9,13]. Also, an imbalance in their production and activation, relative to their inhibition by tissue inhibitors of metalloproteinases (TIMP) can cause IVD degeneration [8]. It has been suggested that MMP-1 has an important role in the degeneration of IVDs [10, 14] and that it is capable of degrading native fibrillar collagens in the ECM [15]. Furthermore, MMP-13 is a key enzyme in the development of osteoarthrtitis [16] and is associated with matrix mineralization [17].

Fragments of type II collagen have been shown to cause inhibition of cell attachment to collagen, inhibition of collagen synthesis, and induction of matrix degradation in cartilage [18]. These collagen fragments are elevated in degenerated discs, being significantly higher in both the AF and the NP than in articular cartilage [19]. Elevated levels of these fragments suggest that disc cells are responding to the altered environment. Type II collagen fragments resulting from normal or enhanced proteolytic activity could be a mechanism that induces the cell to degrade the matrix further.

We now propose to elucidate whether collagen fragments can also induce gene expression in the IVD. Specifically, we tested the ability of the type II collagen fragment, CII (245-270), known to be critical in MHC class II antigen presentation of arthritis patients [20], to modulate the expression of proteinases and proteinase inhibitors, collagen, and aggregan in NP and AF cells of the IVD. This could be of great value in efforts to understand the regulation of collagen cleavage in disc degeneration.

## MATERIALS AND METHODOLOGY

### Source of Tissue

Bovine tails (2-3 years old) were obtained from Les Abattoirs Billette (St-Louis-de- Gonzague, QC, Canada). All IVDs were classified as non-degenerated grade I according to the grading system of Thomson *et al.* [21].

### Cell Isolation

Cells from 10 bovine tails (~15x10^6^ cells from the AF per tail and ~5x10^6^ cells from the NP per tail) were isolated immediately after transportation from the abattoir. The procedures were the same as described before [22]. Briefly, the IVDs were dissected from their adjacent vertebral bodies and placed in medium A, (DMEM-high glucose - 1 µg/ml fungizone). Under aseptic conditions, the IVDs were separated by dissection into regions corresponding to the AF and NP. The AF and NP were dissected into approximately 4-mm thick fragments and were washed twice in medium A for 15 min.

Cells were enzymatically isolated from the tissue using a sequential protease type XIV/collagenase protocol [22]. NP tissues were incubated overnight at 37°C with gentle agitation (250 rpm) in DMEM high glucose supplemented with 10% fetal bovine serum (FBS; HyClone, Logan, UT), 100 U/ml penicillin, 100 μg/ml streptomycin, and 0.08% (w/v) collagenase (Sigma-Aldrich, Oakville, ON, Canada). AF tissues were digested first, for 1h at 37ºC under gentle agitation (250 rpm) in DMEM high glucose supplemented with 10% FBS, 100 U/ml penicillin, 100 μg/ml streptomycin, and 0.4% (w/v) type XIV bacterial protease (Sigma-Aldrich) and then overnight with 0.06% collagenase. The resulting cell suspensions were passed through a 70-μm cell strainer (Becton Dickinson, Oakville, ON, Canada) and washed twice in DMEM high glucose supplemented with 10% FBS, 100 U/ml penicillin, 100 μg/ml streptomycin, and 50 µg/ml L-ascorbic acid (Sigma-Aldrich). Cells were recovered by centrifugation at 400 × *g* for 5 min. Cells were counted in a hemacytometer and the viability was determined using 0.04% Trypan Blue.

### Cell Culture Conditions

Bovine AF and NP cells (1x10^5^ cells in 10 ml medium) were cultured for 2 days in 100 mm dishes in DMEM-high glucose supplemented with 100 U/ml penicillin, 100 µg/ml streptomycin, 2mM glutamine, 50 µg/ml ascorbic acid (prepared fresh), 1mg/ml bovine serum albumin (BSA), 5 µg/ml insulin, 5 µg/ml transferrin, 5 ng/ml sodium selenite. At the time of experiments, the CII (245-270) fragment (US Biological, Swampscott, MA), dissolved in culture media, and was added at final concentrations of 0.1 and 1 μg/ml. These concentrations were chosen from preliminary experiments to reflect very low to high response in gene expression. The cells were maintained in a humidified incubator at 37°C with 5% CO_2_ in air. The medium was changed every two days.

### Total RNA Isolation

Total RNA was extracted from bovine AF and NP cells by a modified method of Chomcynski and Sacchi [23] using TRIzol® reagent (Invitrogen, Burlington, ON, Canada). After centrifugation for 15 min at 12,000 × *g *at 4°C, the aqueous phase was precipitated in 1 volume isopropanol, incubated for 60 min at room temperature, and centrifuged for 15 min at 12,000 × *g* at 4°C. The supernatant was discarded and the RNA pellet was air dried and then resuspended in 50 µl DEPC-treated distilled water. Purity of the RNA was measured through the A_260_/A_280_ ratio.

### Reverse Transcriptase (RT) Reaction

The RT reaction was performed for 50 min at 42ºC using 1.0 µg total RNA. In 20 μl reaction volume, there is 50 mM Tris-HCl (pH 8.3), 75 mM KCL, 3 mM MgCl_2_, 10 mM DTT, 50 μM dNTP mixture, and 200 units of Superscript II RNAse H reverse transcriptase (Invitrogen). The inactivation reaction was achieved at 70ºC for 15 min.

### Polymerase Chain Reaction (PCR)

PCR was performed in a total volume of 25 μl containing: 10 mM Tris-HCl (pH 8.3), 1.5 mM MgCl_2_, 0.2 mM dNTP mixture, 0.8 μM of forward and reverse primers, 1μl of RT mixture, and 1.25 units of Taq DNA polymerase (Invitrogen). PCR procedure was according with the standard method [24, 25]. Thirty cycles were used for all genes. Every cycle consists of 1 min denaturation at 95°C, 1 min annealing at 55°C (COL2, AGG, MMP-1, TIMP-1, TIMP-3, CTSK, IL-6, and IL-1α) or 50°C (MMP-13, TIMP-2, and 18s rRNA), 1min polymerization at 72°C, followed by a final 10 min extension at 72°C. PCR products were separated on 2% agarose gel, visualized by ethidium bromide staining, and analyzed using the Bio-Rad VersaDoc equipped with a cooled charge coupled device (CCD) 12-bit camera (Bio-Rad, Mississauga, Ontario, Canada). 18S rRNA was used as house keeping gene for gel loading and serves to normalize the results. To confirm the lack of genomic DNA contamination of RNA samples, PCR was also performed with RNA aliquots. The primers, specific for bovine genes, were obtained from Invitrogen. The sequences of the primers used for PCR are described in Table **1**.

## RESULTS

### Effect of the CII (245-270) Peptide on the Expression of Aggrecan and Type II Collagen

**Fig. (1):**
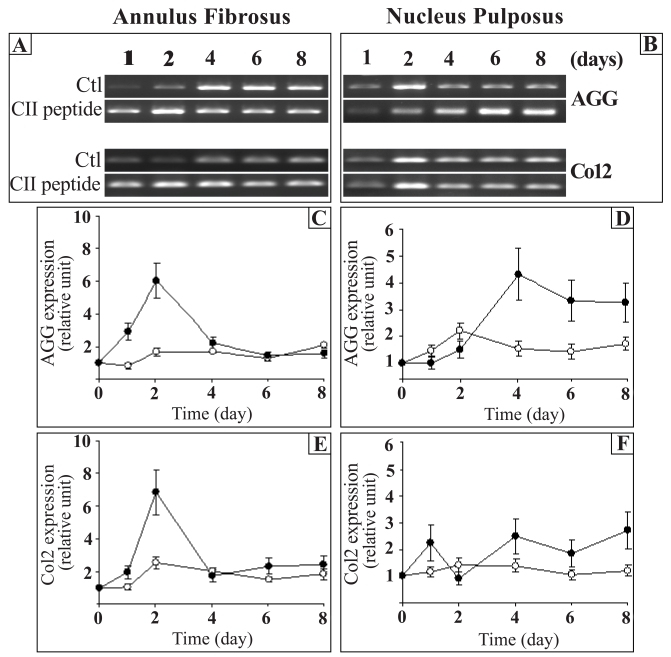
Effect of the CII (245-270) peptide on the gene expression of aggrecan (AGG) and type II collagen (Col2). AF and NP cells were incubated in the absence (Control (Ctl);-○-) or the presence of the CII (245-270) peptide (-●-). Agarose gels are representative of 3 experiments while quantitative results are the mean ± SE of 3 experiments normalized to18S RNA content.

We first examined the expression of aggrecan because the discs resist compressive forces by their high content of the proteoglycans (predominantly aggrecan). Type II collagen was studied because it is important for the integrity of the disc, as it forms the fibrous framework of the tissue. The expression of both aggrecan (AGG) and type II collagen (Col2) was constant throughout the time in culture in both AF and NP cells, except for AGG in the NP where a significant increase was observed after 2 days (Fig. **1B**,**D**). The CII (245-270) peptide strongly up-regulated the expression of AGG on days 1 and 2 in the AF cells (3.7 times the control) while its expression returned to control levels thereafter (Fig. **1A**,**C**). Of special interest here was that the CII (245-270) peptide induced the expression of AGG only after 4 days in NP (2.8 times the control). This increase was maintained throughout the time in culture (Fig. **1B**,**D**). This is in contrast to AF where the presence of the peptide induced AGG expression only at early time points. Results also showed that the CII (245-270) peptide enhanced the expression of Col2 in a time-dependant manner with maximal stimulation reached after 2 days in culture (2.7 times the control) and a return to control level thereafter in AF cells (Fig. **1A**,**E**). In NP cells, the CII (245-270) peptide had a slight up-regulation of Col2 with a maximal stimulation reaching 1.8 times the control after 4 days in culture (Fig. **1B**,**F**).

At a lower concentration (0.1 µg/ml), the CII (245-270) fragment had no effect on the expression of these genes (results not shown).

### Effect of the CII (245-270) Peptide on the Expression of Proteinases

**Fig. (2):**
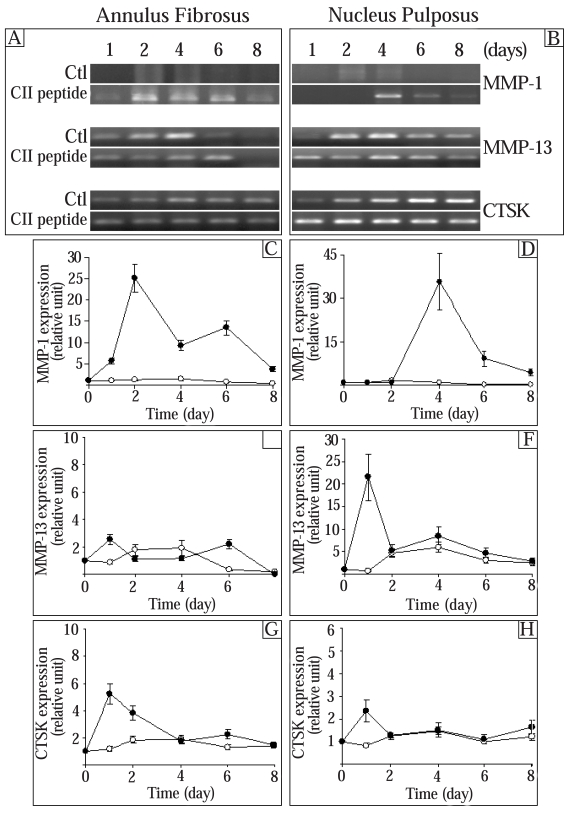
**Effect of the CII (245-270) peptide on the expression of proteinases.** AF and NP cells were incubated in the absence (Control (Ctl);-○-) or the presence of the CII (245-270) peptide (-●-). Agarose gels are representative of 3 experiments while quantitative results are the mean ± SE of 3 experiments normalized to 18S RNA content.

Since MMP-1, MMP-13, and cathepsin K (CTSK) have been implicated in the breakdown of the disc ECM [8-14], we next tested the effect of the CII (245-270) peptide on these proteinases. The control MMP-1, MMP-13, and cathepsin K (CTSK) expression levels were constant throughout the time in culture in both AF and NP cells (Fig. **2**). The expression of MMP-1 was enhanced by the CII (245-270) peptide in AF and NP cells in a time-dependent manner with maximal effect after 2 days for AF cells and after 4 days for NP cells (Fig. **2C**,**D**). The stimulation decreased thereafter. MMP-13 gene expression had a tendency to decrease in AF cells at 6 and 8 days (Fig. **2E**) while it tended to increase with time in NP cells (Fig. **2F**). Except for day 1 in NP cells when MMP-13 was increased (30 times the control), CII (245-270) peptide had very little effect on the expression of MMP-13 (Fig. **2E**,**F**). Finally, addition of the CII (245-270) peptide led to a time-dependent increase of CTSK expression in AF cells with a maximal stimulation reached after 1 day in culture (2.9 times the control) and a decrease thereafter to reach control levels after 4 days in culture (Fig. **2G**). In NP cells, the CII (245-270) peptide stimulated CTSK expression only after 1 day in culture (3.0 times the control) (Fig. **2H**).

At a lower concentration (0.1 µg/ml), the CII (245-270) fragment had no effect on the expression of these genes (results not shown).

### Effect of the CII (245-270) Peptide on the Expression of Tissue Inhibitor of Metalloproteinases (TIMPs)

**Fig. (3):**
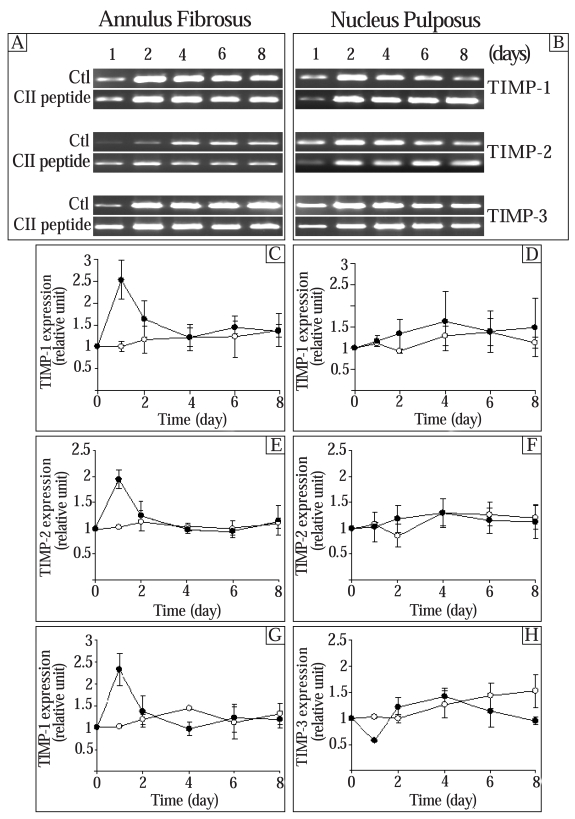
Effect of the CII (245-270) peptide on the expression of tissue inhibitor of metalloproteinases (TIMPs). AF and NP cells were incubated in the absence (Control (Ctl);-○-) or the presence of the CII (245-270) peptide (-●-). Agarose gels are representative of 3 experiments while quantitative results are the mean ± SE of 3 experiments normalized to 18S RNA content.

Since MMPs are inhibited by specific endogenous tissue inhibitor of MMPs (TIMPs) [8], we next determined the effect of the CII (245-270) on TIMP-1, TIMP-2, and TIMP-3. The expression of control TIMP-1, TIMP-2, and TIMP-3 generally had a tendency to increase (not significantly) throughout the culture time (Fig. **3**). In AF cells, the expression of TIMP-1 was stimulated by the CII (245-271) peptide after 1 day in culture and returned to control levels thereafter (Fig. **3C**). The CII (245-270) peptide had no significant effect on TIMP-1 gene expression in NP cells (Fig. **3D**). Addition of the CII (245-270) peptide resulted in a significant increase of TIMP-2 expression after 1 day in culture in AF cells (1.9 times the control) (Fig. **3E**). The expression of TIMP-2 returned to control levels thereafter. In NP cells, the CII (245-270) peptide had no effect on the expression of TIMP-2 at the incubation times we studied (Fig. **3F**). The addition of the CII (245-270) peptide to AF cells led to a significant increase of TIMP-3 after 1 day in culture (2.3 times the control) and a 44% inhibition of its expression after 4 days in culture (Fig. **3G**). In NP cells, the CII (245-270) peptide decreased the expression of TIMP-3 after 1 day (56% of control) and 8 days (63% of control) in culture whereas it had no effect on days 2, 4 and 6 (Fig. **3H**).

At a lower concentration (0.1 µg/ml), the CII (245-270) fragment had no effect on the expression of these genes (results not shown).

### Effect of the CII (245-270) Peptide on the Expression of Interleukins (ILs)

**Fig. (4):**
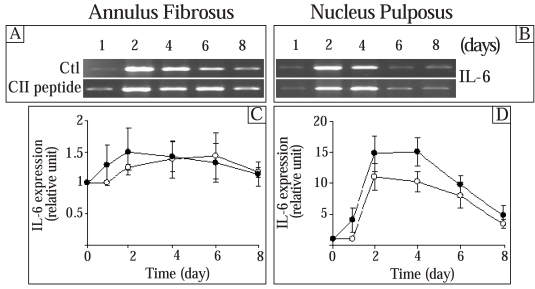
Effect of the CII (245-270) peptide on the expression of IL-6. AF and NP cells were incubated in the absence (Control (Ctl);-○-) or the presence of the CII (245-270) peptide (-●-). Agarose gels are representative of 3 experiments while quantitative results are the mean ± SE of 3 experiments normalized to 18S RNA content.

Since cytokines are implicated in the ECM remodeling [26, 27] and IL-6 has been reported as an important cytokine in the IVD [28], we next examined the effect of the CII (245-270) peptide on the expression of IL-6 and IL-1α. The control IL-6 expression was not statistically different throughout the culture time in AF cells while a significant increase was observed in NP cells on day 2 (Fig. **4**). A progressive decrease of this control level was observed thereafter. In AF cells, the CII (245-270) peptide had no significant effect on the expression of IL-6 (Fig. **4C**). In NP cells, addition of the CII (245-270) peptide led to a time-dependent increase in IL-6 expression with maximal stimulation reached after 4 days in culture (1.4 times the control) (Fig. **4D**). The expression of IL-6 returned to control levels thereafter. At a lower concentration (0.1 µg/ml), the CII (245-270) fragment had no effect on the expression of this genes (results not shown). Furthermore, IL-1α was neither expressed nor stimulated by the CII (245-270) peptide in these cells (results not shown).

### Effect of the CII (245-270) Peptide on MG-63 Osteoblast-Like Cells

MG-63 osteoblast-like cells, cultured as previously described [29], were used as control cells of non-IVD origin. The results show that the CII (245-270) peptide had no effect on these cells, both at 0.1 and 1 µg/ml (results not shown).

## DISCUSSION

There is increasing evidence that once fragmentation of matrix molecules occurs around the disc, degradation products may further enhance matrix degradation [30, 31]. However, there has been no satisfactory explanation on the role of type II collagen fragments in disc degeneration, nor are there many details on how they affect collagenase expression. MMPs play an important role in the IVD but have received sparse attention in the literature [8]. The clinical significance is that knowledge of these feedback mechanisms, in which elevated concentrations of denatured collagens may play a role in the excessive resorption of matrix molecules observed in disc degeneration, may provide new therapeutic targets for regulation of disc destruction in degenerative disc disease. This field of application is of particular interest because conventional treatments are disappointing in chronic low back pain. In this regard, it has been shown that type II collagen fragments inhibited cell attachment to collagen and collagen synthesis, and induced matrix degradation in cartilage [18]. Furthermore, it has been shown that the CII (245-270) fragment can induce arthritis in an animal model [32]. This peptide is the product of the cleavage of Col2 by cyanogen bromide and may not exist in nature, even if it has arthritogenic potential [20]. However, similar peptides are present in the synovial fluid from which they enter the lymphatics and drain eventually into peripheral blood [33, 34]. It is also important to note that collagenase-cleaved collagens unwind and become susceptible to further degradation by other proteinases in the extracellular space [35]. Nevertheless, the present results suggest that degradation products of collagen may be directly involved in the regulation of IVD homeostasis. However, the CII (245-270) peptide had no significant effect on the proliferation of AF and NP cells (results not shown).

IVD and cartilage matrix degradation generates type II collagen fragments [18]. The objective of this study was to explore the possibility that these collagen fragments may be part of an endogenous metabolic feedback loop. The concept of endogenous metabolic feedback loop is supported by the finding that the CII (245-270) peptide can alter the expression of the key ECM genes like AGG and Col2. The stimulation of MMP-1 in AF and NP cells, of MMP-13 in NP cells and of CTSK in AF cells also suggests that the CII (245-270) peptide stimulates a more generalized remodeling in disc cells. This is supported by the effect of the CII (245-270) peptide on TIMP-1, TIMP-2, and TIMP-3 in AF and NP cells.

It is also worthy of note that the CII (245-270) fragment generally had a more pronounced effect on the expression of genes in AF cells than in NP cells, suggesting specificity in the effect of the CII (245-270) fragment. The specificity of the CII (245-270) peptide is also supported by the absence of effect of the CII (245-270) peptide on MG-63 osteoblast-like cells. Moreover, the very high effect on AGG in AF cells (30 times the control) and the prolonged effect on MMP-13 in NP cells, which are different to that observed with the other genes, support a gene and tissue specificity of the CII (245-270) peptide effects. In fact, proteolytic damage of collagens increases with age and becomes more extensive from the outer AF to the NP. The accumulation of such damaged collagen would be eventually expected to weaken the mechanical strength of the tissue, possibly adversely affecting the behavior of other spinal structures such as muscles and ligaments. Thus, the design of therapeutic targets for regulation of disc degeneration is important clinically because there is an association of IVD degeneration with back pain. However, it is difficult to be sure that AF and NP cell phenotypes are maintained throughout the time of culture since currently no universally accepted markers of these cells exist. It was for example demonstrated that there is significant reduction in Col2 and AGG mRNA in NP cell culture compared to fresh NP tissue, although the mRNA amounts remain relatively constant after 2-4 passages [36]. Therefore, the long-term effects of the CII (245-270) peptide on the expression of the different genes (return to control level or sustained expression) may also be due to phenotype changes, due to cell culture conditions and/or the presence of the CII (245-270) peptide. This remains to be investigated.

It was suggested that cytokines are implicated in the ECM remodeling [26, 27]. In the present study, we showed that the CII (245-270) peptide had very weak effect on IL-6 expression, suggesting that this cytokine is not implicated in the effect of collagen fragments. This suggests that IL-6 may be more important in response to pro-inflammatory stimulus such as lipopolysaccharide [37] or in presence of macrophages [38] than in the feedback effect of the CII (245-270) fragment on matrix turnover in IVD tissue. The effect of other collagen fragments on IL-6 expression remains to be investigated. Finally, the absence of IL-1α expression in both AF and NP cells is in agreement with previous studies showing the expression of this cytokine in only 39% of patients with herniated lumbar IVDs [39] and the fact that IL-6 has been reported as the more significant cytokine in the IVD [28].

## CONCLUSIONS

Taken together, our results suggest that collagen fragments from the ECM are capable of interacting with cells from the IVD and may regulate many biological processes important to disc homeostasis, degeneration or repair. The expression of genes resulting from these interactions may vary in intensity and specificity, depending on the cell type. Thus, the present study highlights the importance of understanding cell matrix interactions at the molecular level. Indeed, the effect of the CII (245-270) peptide on other ECM related genes (collagens, MMPs, TIMPs, etc.), the effect of other peptides produced by cyanogen bromide and generating T-cells response (CII (189-209) peptide) [20], and the existence of receptors for these peptides on AF and NP cells is yet to be established.

## Figures and Tables

**Table 1.** Primer Sequences for the Study of Gene Expression

Gene	Primer Sequence	Size of the PCR Product
AGG	Forward (721-740): 5’-CAGAACATGCGC TCCAATGA-3’ Reverse (1071-1090): 5’-CGTCATAGGTTTCGTTGGTG-3’	370 bp
COL2	Forward (949-970): 5’-GAACCCAGAAACACAATCC-3’ Reverse (1075-1095): 5’-GTTCGGACTTTTCTCCCCTC-3’	147 bp
MMP-1	Forward (26-46): 5’-TCT GCT GCT GCT GCT ACT CTG-3’ Reverse (400-419): 5’-TTT CTC AAT GGC TTG GTC CA-3’	394 bp
MMP-13	Forward (1237-1255): 5’-GATAAAGACTATCCGAGAC-3’ Reverse (1365-1382): 5’-CGAACAATACGGTTACTC-3’	146 bp
CTSK	Forward (88-107): 5’-AAGAAGACCCACAGGAAGCA-3’ Reverse (537-558): 5’-AAGGCATTGGTCATGTAGCC-3’	471 bp
TIMP-1	Forward (377-394): 5’-GACAATTGTCGAATGGGC-3’ Reverse (716-740): 5’-CTGTTTCCACTCCCACCTTTTTTTC-3’	364 bp
TIMP-2	Forward (325-342): 5’-GAGTATCTCATTGCAGGG-3’ Reverse (480-500): 5’-CAT GATCCCATGCTACATCTC-3’	176 bp
TIMP-3	Forward (181-200): 5’-GTCTACACCATCAAGGAGAT-3’ Reverse (544-564): 5’-TTCTGCCGGATGCAAGCGTA-3’	384 bp
IL-6	Forward (1-19): 5’-ATGAACTCCCGCTTCACA-3’ Reverse (603-623): 5’-TTCATCCGAATAGCTCTCAGG-3’	623 bp
IL-1α	Forward (126-146): 5’-ACTTCGTGAGGACCAGATGAA-3’ Reverse (746-765): 5’-ACTTGCCATGTGCACCAATT-3’	640 bp
18S RNA	Forward (153-176): 5’-CTA CTT GGA TAA CTG TGGTAA TTC-3’ Reverse (305-321): 5’-GAC TCT AGA TAA CCT CG-3’	169 bp
